# Molecular identification and antifungal susceptibility profiles of etiologic agents of oral candidiasis among HIV-positive patients: A multicenter study

**DOI:** 10.18502/CMM.2023.345058.1414

**Published:** 2023-06

**Authors:** Hamid Morovati, Malihe Jokari, Saba Eslami, Kamiar Zomorodian, Katayoun Taeri, Nesa Khalaf, Hossein Khodadadi

**Affiliations:** 1 Department of Parasitology and Mycology, School of Medicine, Shiraz University of Medical Sciences, Shiraz, Iran; 2 Central Research Laboratory, School of Medicine, Shiraz University of Medical Sciences, Shiraz, Iran; 3 Basic Sciences in Infectious Diseases Research Center, Shiraz University of Medical Sciences, Shiraz, Iran; 4 Behavioral Disease Council Center, Isfahan University of Medical Sciences, Isfahan, Iran; 5 Department of Molecular Medicine, School of Advanced Medical Sciences and Technologies, Shiraz University of Medical Sciences, Shiraz, Iran

**Keywords:** Drug resistance, HIV, Oral candidiasis, RFLP

## Abstract

**Background and Purpose:**

Human immunodeficiency virus (HIV)/acquired immunodeficiency syndrome (AIDS) is a serious risk factor for oral candidiasis (OC). In this regard, the present study aimed to investigate the frequency of *Candida* species collected from the oropharyngeal cavity of HIV-positive patients and the sensitivity of these isolates to antifungal drugs.

**Materials and Methods:**

Oral samples were collected from 169 HIV-positive patients. In addition to culture-based methods, a molecular assay via the polymerase chain reaction-restriction fragment length polymorphism method was applied to identify isolates using the *MspI* restriction enzyme. The disk diffusion method determined the susceptibility of isolated yeasts to common antifungal drugs according to the CLSI M44-A2 protocol.

**Results:**

In total, 81 participants (47.92%) were positive for OC, and *Candida albicans* was the most prevalent yeast (53.98%). The median age of patients was 36 years old (IQR=10.5; 17-59), and it was found that women are 27% more susceptible to HIV-associated OC (OR=1.268; 95% CI: 0.685-2.348). Patients who received antifungal therapy had a 97.3% reduced chance for OC (OR: 0.027; 95% CI: 0.008-0.091; *P-value*: 0.000). Antifungal therapy reduced the risk of OC by 97.3% (OR=0.027; 95% CI=0.008-0.091; *P*=0.000), and antiretroviral therapy decreased the chance of OC 4.42 times (OR=4.423; 95% CI=1.697-11.528; *P*=0.002). The resistance rates for antifungals, namely fluconazole, ketoconazole, itraconazole, amphotericin B, and nystatin were 15.93%, 8.85%, 7.96%, 5.31%, and 4.42%, respectively.

**Conclusion:**

Although several decades have passed since the emergence of HIV/AIDS, little information is available about fungal colonization and infections in this population. Further investigations are suggested using novel and reference molecular identification methods, such as matrix-assisted laser desorption ionization time-of-flight mass spectrometry and sequencing, respectively. In addition, more reliable methods for antifungal susceptibility testing are recommended.

## Introduction

Candidiasis comprises a wide spectrum of opportunistic fungal infections with various clinical forms caused by *Candida* species [ 1 ]. Oral candidiasis (OC) remains one of the most important clinical manifestations of the Human immunodeficiency virus (HIV) setting [ 2 , 3 ]. The HIV/acquired immunodeficiency syndrome (AIDS) is a serious risk factor for several fungal infections, mainly OC [ 4 ]. *Candida albicans* is the predominant etiologic agent for OC (40-75%), but its balance with non-*albicans Candida* strains has been changing in the last two decades [ 5 ]. More than 90% of HIV patients present OC, especially in the early, pre-treatment, and advanced stages of HIV/AIDS [ 6 , 7 ]. Up to 50% of the untreated population and 90% of HIV/AIDS individuals have OC, depending on the cohort examined and the stage of HIV/AIDS infection [ 6 - 8 ]. Although OC is not a specific sign of HIV/AIDS, in most cases, it can be a sign of a weakened immune system in the final stages of HIV/AIDS [ 6 - 8 ]. Colonized *Candida* species OC may switch to invasive fungal infection (IFI), which is a significant cause of death among uncontrolled HIV/AIDS patients [ 9 , 10 ].

Despite widespread antiretroviral therapies (ART), IFIs cause one million deaths each year, 50% of which are related to HIV/AIDS patients [ 2 , 11 ]. Therefore, isolation, identification, and definition of drug-resistance patterns of the causative agents are essential for the selection of the appropriate treatment tool. It should be noted that the clinical findings are the first alarm for OC. Moreover, molecular techniques, such as conventional polymerase chain reaction (PCR), multiplex PCR, PCR-restriction fragment length polymorphism (RFLP), and real-time PCR can be used to detect causative agents [ 12 , 13 ]. There are several antifungal therapy (AFT) options for OC among the HIV-positive population [ 14 ]. Triazoles are the first line of prophylactic and empirical treatment for OC [ 15 ]. However, it is concerning that prolonged and repetitive treatment has resulted in triazole-resistant isolates among *Candida* species [ 16 , 17 ]. 

This study investigated the prevalence, molecular identification, and antifungal susceptibility profiles of *Candida* species colonized in the oral cavities of HIV-positive patients in Iran. The results of this study may pave the way for the management and control of OC among HIV/AIDS patients.

## Materials and Methods

### Patients

This multicenter cross-sectional observational study was conducted from December 2018 to May 2020. In total, 169 HIV-positive patients who were referred to the Behavioral Diseases Centers in Tehran, Isfahan, Fars, and Bushehr provinces in Iran were enrolled in this study. The HIV-positive patients with proven oral manifestation of Candidiasis were included in this study. Patients who did not have a clearly defined status of receiving ART and AFT were excluded from this study. The age, gender, underlying diseases, and smoking or alcohol consumption status of the patients were recorded if available. Furthermore, CD4^+^ T cells were counted in patients using the CD4 cytometer (portable or bench-top CyFlow® miniPOC Sysmex Partec GmbH). The OC was approved according to the diagnostic criteria for candidiasis, as published by the Centers for Disease Control and Prevention (CDC).

### Samples and initial yeast isolation

Clinical samples were collected via sterile swabs from 169 patients (one sample per patient) and immediately cultured on Sabouraud dextrose agar (SDA) (Merck, Germany) containing antibiotics (chloramphenicol or penicillin-streptomycin) and were incubated at 32 °C for 72 h. Yeast-positive samples (n=81) were transferred to sterile Eppendorf tubes containing sterile normal saline, packed in a cool box, and then sent to the referral laboratory of medical mycology at the School of Medicine in Shiraz, Iran.

### Screening of Candida species

A loop of each Eppendorf content was cultured on SDA, as described above. The resulting single colonies were then cultured linearly on HiChrom *Candida* agar plates (HiMedia, India) for morphologic differentiation of Candida species. Each colony was isolated and maintained as an independent sample according to its color. Single colonies were selected from mixed samples containing a mixture of yeasts through several dilutions and successive passages on the SDA. Pure colonies were dissolved in sterile water in Eppendorf tubes and stored at minus 20 °C for molecular assay and antifungal susceptibility testing (AFST).

### Molecular assays

### DNA extraction and Polymerase Chain Reaction

The lithium acetate method with some modifications was applied for DNA extraction [ 18 ]. Quantitative evaluation of extracted DNA by NanoDrop2000 Spectrophotometer (Thermo-Scientific Inc., USA) showed that an average of 17.6 ng (ng/µl) of DNA was extracted from each sample. The *ITS1-5.8S* -*ITS2* gene region was targeted for amplification via panfungal ITS1/ITS4 primer pairs (ITS1: 5'- TCCGTAGGTGAACCTGCGG-3'; ITS4: 5'- TCCTCCGCTTATTGATATGC-3'). The PCR conditions were as follows: denaturation phase 1 cycle at 94 ℃ for 10 min followed by 35 cycles at 94 ℃ for 45 s, 56 ℃ for 45 s, and 72 ℃ for 1 min, and a final extension at 72 ℃ for 7 min. In the final step, the quality of PCR products was controlled using agarose gel electrophoresis by the Gel Doc XR system (Biorad, USA) and 100 bp DNA size marker (GenetBio, Korea) [ 19 ]. The positive and negative controls were 10 ng/μl of DNA *C. albicans* ATCC 10231 and sterile distilled water, respectively.

### Polymerase chain reaction-restriction fragment length polymorphism

The PCR-RFLP was performed according to the previously approved protocol introduced by Mirhendi et al. [ 20 ]. Briefly, ITS1-5.8S-ITS2 PCR products were digested by the *Msp*I enzyme. This enzyme breaks down *Candida* genomic DNA into pieces of different sizes. After completing the RFLP, electrophoresis was performed to observe the created bands. Finally, *Candida* species were identified according to the pattern and size of each band.

### Antifungal susceptibility testing

The AFST for five common antifungals, including amphotericin B (AmB), fluconazole (FLC), itraconazole (ITC), ketoconazole (KTC), and nystatin (NY), was assayed via the disk diffusion method according to the protocol provided by the American Clinical and Laboratory Standards Institute (CLSI M44-A2) [ 21 ]. The accuracy of the diameter of the growth inhibition zone around the discs was checked using standard yeast species in addition to the table provided by the manufacturer (Rosco diagnostica, Denmark). Quality control strains of *C. albicans* (ATCC90029), *C. parapsilosis* (ATCC 2201), and *C. krusei* (ATCC6258) were obtained from ATCC.

### Statistical Analysis

The statistical analysis was performed in the SPSS software (version 24.0). The quantitative variables (age and CD4 count) were tested for normality and mean equality using the Kolmogorov-Smirnov and Mann-Whitney tests, respectively. The qualitative data were compared using the Chi-square approach (gender, AFT, and ART). Besides, the Spearman test was applied to assess the correlation between quantitative variables. The threshold for statistical significance was set at *P* values below 0.05.

### Ethical considerations

All methods were performed in accordance with the relevant guidelines and regulations. This study was supervised and monitored by the Ethics Committee of Shiraz University of Medical Sciences, Shiraz, Iran in 2016 (Permission code: IR.SUMS.REC. 1394.S142). Furthermore, informed consent was obtained from all participants and parents/legally authorized representatives of all minors (below 16 years old) and deceased participants involved in the study.

## Results

### Demographic characteristics of patients

**Figure 1:**
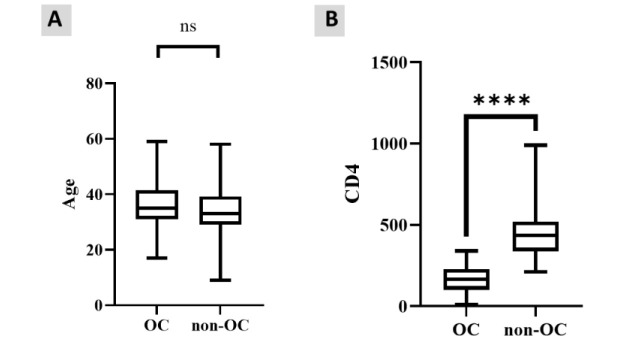
**A, B.** Age range (A: left) and CD4 count range (B: right) of the HIV-positive patients with OC vs. non-OC.

**Figure 2:**
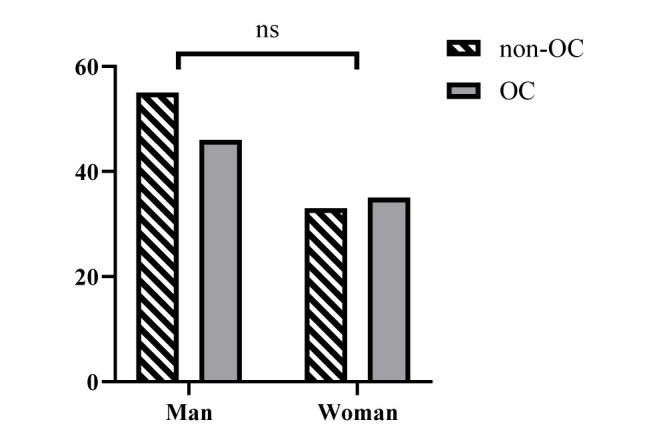
Sex distribution of the HIV-positive patients with OC vs. non-OC.

During this multicenter retrospective survey, a total of 169 HIV patients were studied, and 81 of them (47.92%) were OC-positive. Patients ranged in age from 9 to 59 years old. Details of demographic data are summarized in Supplementary Tables 1 and 2. The Mann-Whitney test revealed that the ages of the patients in the two groups were statistically similar (*P*=0.079) (Figure 1 A). The chi-square test indicated that the gender was not distributed equally among the two groups (*P*=0.572; OR=1.268; 95% CI=0.685-2.348) (Figure 2 and Supplementary Table 2). In total, 52 out of 88 non-OC patients had received AFT, while only three OC-positive patients had received it. This difference was statistically significant (*P*<0.001). 

**Figure 3:**
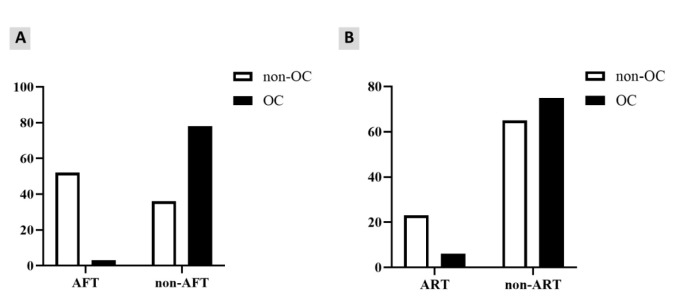
**A, B.** The quantitative status of antifungal therapy (A: left) and antiretroviral therapy (B: right) of the HIV-positive patients with OC vs. non-OC.

Moreover, it was indicated that HIV patients with AFT had a 97.3% reduced chance of OC (OR=0.027; 95% CI=0.008-0.091; *P*<0.001) (Figure 3A and Supplementary Table 2). Seventy-five OC patients and 65 non-OC patients had received ART; however, they were statistically different (*P*=0.002). Besides, it was indicated that ART decreased the chance of OC by 4.42 times (OR=4.423; 95% CI=1.697-11.528; *P*=0.002) (Figure 3B and Supplementary Table 2). Moreover, no statistical relationship was found between age and CD4^+^ T cell count of the patients (Spearman’s ratio=-0.101; *P*=0.192). 

The median of blood CD4^+^ T cell count among OC-positive patients and non-OC patients were 166 cell/µl (IQR=129; 11-340) and 425.5 cell/µl (IQR: 187; 220 to 989), respectively. In addition, the Mann-Whitney test indicated that there was a statistically significant difference between the median CD4 count of OC and non-OC patients (*P*=0.00) (Figure 1B and Supplementary Table 1). In this study, according to Kruskal-Wallis test results, quantitative variables were not distributed normally (Supplementary Figures 1A, 1B, and Supplementary Table 1).

### Microbiological identification of yeasts

From 81 OC-HIV-positive patients, 113 yeast isolates, including 96 (84.95%) *Candida* species plus 17 (15.04%) unknown yeast species, were collected and identified according to culture results on SDA and HiChrom *Candida* agar. The most isolated species was *Candida albicans* (54%). Moreover, 24 out of 81 (29.64%) OC-HIV-positive patients were infected with multiple yeast species (Table 1). 

**Table 1:** Yeast isolates identified by HiChrom *Candida* agar and PCR-RFLP method. NA: not applicable.

Yeast species	RFLP				HiChrom *Candida* agar N (%)
	Frequency (N: 113)	Percentage (N: 110)	Size of PCR Amplicon (bp)	Size of *Msp*I -RFLP fragments (bp)	
***C. albicans***	61 (53.98%)	55.4	537	239, 298	61 (53.98%)
***C. glabrata***	26 (23.01)	23.6	881	320, 561	26 (23.01)
***C. kefyr***	14 (12.39%)	12.7	720	720	NA
***C. tropicalis***	7 (6.19%)	6.36	526	186, 340	7 (6.2%)
***C. krusei***	2 (1.77%)	1.8	510	250, 260	2 (1.87%)
***C. intermedia***	1 (0.88)	0.9	389	122, 267	NA
***C. parapsilosis***	1 (0.88)	0.9	530	530	NA
***C. lusitaniae***	1 (0.88)	0.9	382	118, 264	NA
**Unknown yeast**	0 (0.00%)	0	NA	NA	17 (14.94)

### Yeast identification based on the molecular methods

Following conventional PCR, PCR-RFLP was carried out using the *Msp*I enzyme (Supplementary Figure 2). Results indicated that 81 out of 169 (47.92%) patients tested positive for OC, and 110 *Candida* species were identified in 81 OC-HIV-positive patients. *Candida albicans* was the most identified *Candida* species (54%), and a considerable number of *C. kefyr* species (n=14, 12.7%) was identified (Table 1).

### Antifungal susceptibility testing

In total, 565 AFSTs were carried out (5 antifungals vs. 113 isolates). It was found that 48 were resistant, 70 had an intermediate response, and 447 were susceptible to the five antifungals studied. Among them, most of the isolates were resistant to FLC (n=18, 15.93%), followed by KTC (n=10, 8.85%), ITC (n=9, 7.96%), AmB (n=6, 5.31%), and NY (n=5, 4.42%). In addition, 3 out of 14 (21.42%) of our rarely isolated *C. kefyr* species were resistant to FLC, KTC, and ITC. It should be noted that *C. albicans* was the most resistant species against FLC (14 out of 18, 77.77%). Both *C. krusei* isolates (n=2) were resistant to all antifungals (Supplementary Table 3).

## Discussion

Oral Candidiasis is still the most frequent oral manifestation in HIV-positive patients [ 22 ]. In recent years, both in Iran and around the world, a number of factors that influence the incidence and prevalence of oral candidiasis have changed. The COVID-19 pandemic affected the lifestyles and behaviors of some people. The prevalence of some other infections, such as AIDS or AIDS-related diseases, may be affected by COVID-19 pandemic consequences, such as wearing masks, restricting personal interactions with others, and avoiding risky sexual intercourse. Nevertheless, COVID-19 patients have demonstrated that they are susceptible to a number of various infections. Since COVID-19 and OC have been shown to interact (OR=2.01; 95% CI=1.1870-3.4143, *P*=0.094), its rate could be higher during the COVID-19 pandemic [ 23 ]. Besides, Iran has recently experienced changes in healthcare policies in addition to some economic difficulties.

Some different social and economic variables have affected the incidence and prevalence of STDs and AIDS in Iran during the recent decade. New population rejuvenation programs and rising poverty in Iran have impacted several variables related to the prevalence of OC, particularly those relating to AIDS and STD prevention methods. For instance, restricted access to contraceptives (condoms) for young people, particularly HIV-positive sex workers, has affected the epidemiology of AIDS as well as related infections, such as OC. Additionally, a significant number of refugees from neighboring countries have entered Iran. The epidemiological changes of the affected diseases should occasionally be monitored. However, it should be mentioned that the goal of this study was not to evaluate all of the related OC factors listed above.

The present study was performed on 169 HIV patients, 81 (47.92%) of whom were reported to be OC patients according to clinical signs and symptoms as well as their medical records. According to molecular identification, *C. albicans* (53.98%) was the most prevalent yeast, followed by *C. glabrata* (23.01%), *C. kefyr* (12.39%), *C. tropicalis* (6.19%), *C. krusei* (1.77%), and one isolate of each of *C. intermedia*, *C. parapsilosis*, and *C. lusitaniae* (0.88%). In Iran, Rafat et al. [ 24 ] reported that the pooled prevalence of OC among HIV-infected pediatrics was 23.9% (95% CI=17.3-32.0), and *C. albicans* was the most common agent.

Hosain Pour et al. [ 25 ] declared that *C. albicans* (69.14%), *C. glabrata* (23.46%), *C. parapsilosis* (4.94%), and *C. krusei* (1.24%) were the most predominant agents. In the southwest of Iran, Erfaninejad et al. [ 6 ] estimated a prevalence rate of 41% for HIV-associated OC. They showed that the most common species was *C. albicans* (64.6%), followed by *C. glabrata* (26.5%), and *C. dubliniensis* (19.5%). Moreover, for the first time, they reported one case of each of *C. famata*, *C. africana*, and *C. stellatoidea* isolated from HIV-associated OC patients in this region. One outstanding finding of the present study is the rarely isolated *C. kefyr*. Khedri et al. [ 26 ] and Hosain Pour et al. [ 25 ] reported 7 (6.9%) and 1 (1.24%) *C. kefyr* isolates, respectively. It can be concluded that the prevalence rate and causative agents of OC followed the same pattern in Iran as reported by the mentioned studies. This similar finding may be relevant to the upcoming phase of those studies with us. Similar circumstances may have occurred during the study period. In support of our findings about AFT and ART, Tawerne-Ghadwal et al. [ 5 ] reported that the prevalence of OC among ART-untreated Chadian HIV-positive patients was obviously higher than that among ART-treated patients (16% vs. 2%, *P*<0.01). This indicated the importance of highly active antiretroviral therapy (HAART) to impede OC among this population. Rafat et al. [ 24 ] indicated that ART was meaningfully linked to a reduction in oral *Candida* colonization or infection. Du et al. [ 27 ] reported that the prevalence of OC decreased after HAART initiation (*P*<0.05). Findings of the present study showed that 15.93% of the *Candida* isolates were resistant to FLC. The resistance rates for KTC, ITC, AmB, and NY were 8.85%, 7.96%, 5.31%, and 4.42%, respectively. Moreover, *Candida albicans* was the most resistant species to FLC (14 out of 18, 77.77%). In addition, 3 out of 14 (21.42%) of our rarely isolated *C. kefyr* species were resistant to FLC, KTC, and ITC. 

An outstanding finding was that *C. krusei* isolates (n=2) were resistant to all antifungals. We began by investigating contradictory findings by other researchers. According to Tawerne-Ghadwal et al. [ 5 ], azole antifungal resistance was only observed in *C. krusei* and *C. glabrata*, which are intrinsically resistant species. Ambe et al. [ 28 ] reported that the isolates were mostly susceptible to NY (83.6%), while they were mostly resistant to KTC (29.2%), followed by FLC (24.6%). In addition, Khedri et al. [ 26 ] reported that all *Candida* isolates were susceptible to AmB and CSP, while 16 *C. albicans* (17.6%), 1 *C. dubliniensis*, and 1 *C. glabrata* were resistant to FLC and also Five C. albicans (5.9%) and one *C. tropicalis* was resistant to VRC. Goulart et al. [ 29 ] reported lower rates and declared that resistance to FLC, KTC, and ITC corresponded to 1%, 4%, and 4% of their *Candida* isolates, respectively.

In support of our findings, Rajadurai et al. [ 30 ] performed a network meta-analysis of antifungal effectiveness and concluded that FLC was the most effective antifungal for the treatment of adults with HIV-associated OC, followed by PSC and ITC. However, they stated that PSC is the most effective antifungal for the mycological cure, followed by FLC and that NY was ranked the safest antifungal agent. Even though there were some changes in the antifungal susceptibility results of the compared studies, the patterns were not noticeably different.

Therefore, it seems that the present antifungal prescribing regimens for the management of OC might still be used. Similar to earlier research that found median CD4^+^ T cell counts to be a significant predictor of OC in HIV-positive patients (OR=4.365; 95% CI=1.73-10.98; *P*=0.002), our findings demonstrated that blood CD4^+^ T cell count can also serve as a reliable indicator of OC in HIV-positive patients [ 6 ]. 

The first limitation of the present study was the method of AFST. The disk diffusion method was carried out to check the susceptibility profiles of our isolates to only five antifungals. Another limitation was that the identities of our isolates were not confirmed by the reference sequencing method.

Regarding the strengths of this study, the current epidemiology of oral candidiasis in HIV-positive patients in various parts of the nation was reported simultaneously. Another one of the strengths of this study may be the evaluation of the related OC predisposing factors, such as CD4^+^ T-cell count, in the HIV/AIDS population.

## Conclusion

The prevalence rates of non-albicans *Candida* species are increasing. In addition, resistance rates remained outstanding, especially in *C. glabrata* and the rarely isolated *C. kefyr*. Moreover, CD4^+^ T cell count can be a reliable predictor for OC among HIV-positive patients. Although several decades have passed since the emergence of HIV/AIDS, little information is available about fungal colonization and infections in this population. Therefore, there is an urgent need to screen and follow up on this population. Further investigations are recommended using novel and reference molecular identification methods, such as matrix-assisted laser desorption ionization time-of-flight mass spectrometry and sequencing, respectively. Besides, more reliable methods for AFST are recommended.
